# Rapid Identification of a Novel Complex I *MT-ND3 *m.10134C>A Mutation in a Leigh Syndrome Patient

**DOI:** 10.1371/journal.pone.0104879

**Published:** 2014-08-12

**Authors:** David K. Miller, Minal J. Menezes, Cas Simons, Lisa G. Riley, Sandra T. Cooper, Sean M. Grimmond, David R. Thorburn, John Christodoulou, Ryan J. Taft

**Affiliations:** 1 Queensland Centre for Medical Genomics, Institute for Molecular Bioscience, University of Queensland, St Lucia, Queensland, Australia; 2 Genetic Metabolic Disorders Research Unit, Kids Research Institute, Children’s Hospital at Westmead, Sydney, Westmead New South Wales, Australia; 3 Discipline of Paediatrics and Child Health, Sydney Medical School, University of Sydney, Camperdown New South Wales, Australia; 4 Institute for Molecular Bioscience, University of Queensland, St Lucia, Queensland, Australia; 5 Institute for Neuroscience and Muscle Research, Children’s Hospital at Westmead, Sydney, New South Wales, Australia; 6 Murdoch Childrens Research Institute and Victorian Clinical Genetics Services, Royal Children’s Hospital, Flemington Road, Parkville, Melbourne, Victoria, Australia; 7 Department of Paediatrics, University of Melbourne, Melbourne, Victoria, Australia; 8 Discipline of Genetic Medicine, Sydney Medical School, University of Sydney, Camperdown, New South Wales, Australia; 9 Departments of Integrative Systems Biology and Pediatrics, George Washington University School of Medicine, Washington, D.C., United States of America; University of Sheffield - MRC Centre for Developmental and Biomedical Genetics, United Kingdom

## Abstract

Leigh syndrome (LS) is a rare progressive multi-system neurodegenerative disorder, the genetics of which is frequently difficult to resolve. Rapid determination of the genetic etiology of LS in a 5-year-old girl facilitated inclusion in Edison Pharmaceutical’s phase 2B clinical trial of EPI-743. SNP-arrays and high-coverage whole exome sequencing were performed on the proband, both parents and three unaffected siblings. Subsequent multi-tissue targeted high-depth mitochondrial sequencing was performed using custom long-range PCR amplicons. Tissue-specific mutant load was also assessed by qPCR. Complex I was interrogated by spectrophotometric enzyme assays and Western Blot. No putatively causal mutations were identified in nuclear-encoded genes. Analysis of low-coverage off-target mitochondrial reads revealed a previously unreported mitochondrial mutation in the proband in *MT-ND3* (m.10134C>A, p.Q26K), a Complex I mitochondrial gene previously associated with LS. Targeted investigations demonstrated that this mutation was 1% heteroplasmic in the mother’s blood and homoplasmic in the proband’s blood, fibroblasts, liver and muscle. Enzyme assays revealed decreased Complex I activity. The identification of this novel LS *MT-ND3* variant, the genomics of which was accomplished in less than 3.5 weeks, indicates that rapid genomic approaches may prove useful in time-sensitive cases with an unresolved genetic diagnosis.

## Introduction

Leigh syndrome (LS) is a rare progressive neurodegenerative disorder that is characterised by early onset (typically infancy or early childhood) and a combination of signs including muscular hypotonia or spasticity, dystonia, nystagmus, psychomotor retardation and occasionally epilepsy [1]. LS patients may show rapid loss of previously acquired skills, failure to thrive and feeding difficulties, with survival generally measured in years [1], [2]. Bilateral symmetrical lesions in the central nervous system, particularly in the brain stem, thalamus, and spinal cord are diagnostic, as are findings of lactic acidosis and a Magnetic Resonance Spectroscopy (MRS) lactate peak [1].

LS can be caused by a heterogeneous array of mitochondrial or nuclear genetic mutations that decrease aerobic energy production. Although the most prevalent LS mutations are associated with Complex IV (cytochrome C oxidase complex; e.g. *COX15*, *SURF1*) [OMIM 603646, OMIM 185620], Complex I subunit mutations are also common. Damaging variants have been identified in the mitochondrial genome encoded genes *MT-ND1*, *MT-ND2*, *MT-ND3*, *MT-ND4*, *MT-ND5*, *MT-ND6* [3], [4], and ten nuclear encoded *Complex I* NADH-ubiquinone oxidoreductase (NDU) proteins [5]. LS is the most common clinical presentation of a Complex I deficiency [6], [7].

A clinical diagnosis of LS can be reached without knowledge of the causal mutation, the identification of which, until recently, has often been impractical due to the technical limitations and cost associated with Sanger sequencing multiple nuclear and mitochondrial candidate genes. Here we present the case of a child who required a definitive genetic diagnosis for inclusion in an ongoing Edison Pharmaceutical Phase 2B clinical trial of EPI-743, a para-benzoquinone that facilitates repletion of reduced intracellular glutathione and shows promise for LS patients. All ten LS children participating in a recent Phase 2A EPI-743 study exhibited reversal of disease progression regardless of genetic determinant or disease severity [8]. To identify the causal genetic variant responsible for LS in the proband we employed tiered set genomic investigations across all six members of the immediate family, including development of a targeted Nextera-based mitochondrial DNA sequencing assay, which led to the discovery a novel LS *MT-ND3* mutation.

## Patients and Methods

### Case Report

**Figure 1:**
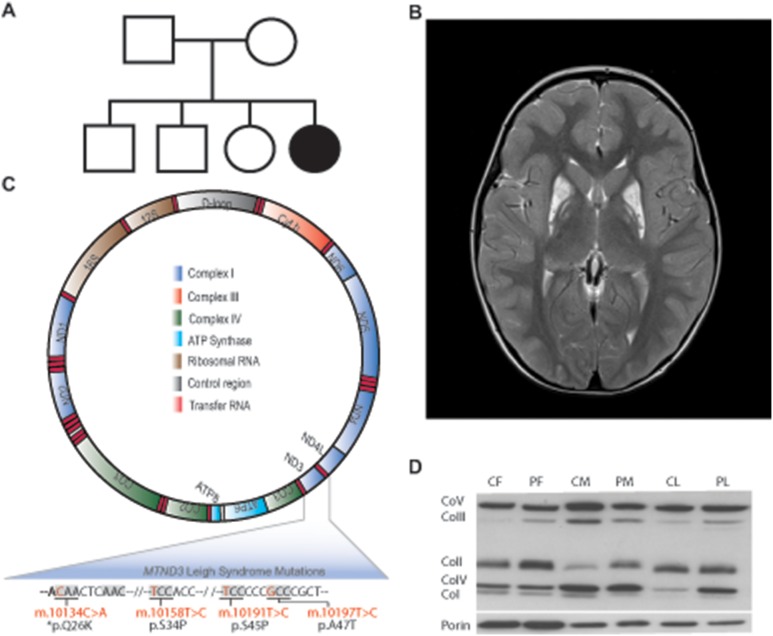
A m.10134C>A Leigh syndrome mutation. (**A**) Family pedigree with the LS patient annotated as a filled black circle. (**B**) T2 supra-orbital MRI of the proband showing symmetrical bilateral cystic gliosis within the globus pallidus (orange arrows). (**C**) Graphical depiction of the mitochondrial genome, annotated with the *MT-ND3* LS mutation identified in this study (bottom left, highlighted with an asterisk) and three previously described mutations (see text for references). (**D**) Western blot analysis of Complex I components, where CF and PF denote to control and patient fibroblasts), CM and PM denote control and patient muscle biopsy samples, and CL and PL denote control and patient liver biopsy samples. These data show a reduction of Complex I (CoI) protein levels in muscle, but no significant reduction in liver, consistent with the results shown in Table 2. We also observed elevated Complex II (CoII) in patient fibroblasts and muscle, Complex III (CoIII) in patient fibroblasts and liver, and Complex IV (CoIV) in patient liver, suggesting compensatory mitochondrial proliferation.

The proband, a girl, is the youngest of four healthy children born to unrelated and clinically unremarkable Australian parents (Figure 1A). There were no antenatal or postnatal issues of note, and early growth and development were normal. First concerns were raised when the proband was approximately 4 years of age, when it became apparent that she had lost her ability to jump and had developed an abnormal gait. It was also noted at this time that her speech development was delayed. The proband’s parents stated that she was slower in acquiring new skills than her siblings from an early age. Following an MRI/MRS of the brain under general anaesthesia at 4.5 years of age her speech became more difficult to understand, her mobility and balance worsened, and her behaviour deteriorated. Formal cardiology, audiology and ophthalmology assessments were normal.

The patient’s score on the Newcastle Paediatric Mitochondrial Disease Scale for sections I-III was 17 (range 0–79). Diagnostic investigations were consistent with LS. The brain MRI revealed extensive signal abnormality involving putamen, and to a lesser extent, the globus pallidus bilaterally and the cerebral peduncles (Figure 1B). MRS also revealed elevated lactate in the basal ganglia (data not shown), although blood and CSF lactate levels were normal. Liver function enzymes were all within the normal range. The muscle biopsy showed fat accumulation and slightly increased subsarcolemmal mitochondrial aggregates. However there were no ragged red fibres and COX/SDH immunohistochemical staining was normal. Histological studies on the liver biopsy showed an increased glycogen content and mild biliary ductular proliferation, but no fibrosis. Electron microscopy study of the liver showed occasional moderately enlarged mitochondria with paracrystalline and crystalline inclusions.

### Genomics and Bioinformatics

#### High-density SNP Arrays & Exome Sequencing

This research was approved by the Human Research Ethics Committees of the Children’s Hospital at Westmead and the University of Queensland, and written consent was obtained from the parents on behalf of themselves and their children (all children aged less than 10 years), as approved by the Human Research Ethics Committees. DNA was extracted from peripheral blood samples from the patient, both parents and three unaffected siblings. Copy number changes (e.g. large indels) and genomic rearrangements were assessed using the Illumina HumanOmni2.5-8 Multi-Use SNP arrays. Exome sequencing libraries were prepared with the TruSeq DNA sample prep kit, and exome capture was performed using the Nimblegen v3 Human EZ-Exome kits. Libraries from all six individuals were generated within 96 hours of sample receipt. Rapid sequencing (including on-instrument cluster generation) was performed on the Illumina HiSeq 2500 in less than twenty-seven hours.

#### Targeted Mitochondrial Sequencing

Three primers pairs [9] were used to generate overlapping PCR products (each ∼6,000 bp) that encompassed the entire mitochondrial genome. PCR products from each member of the family were used to create libraries compatible with MiSeq and Ion Torrent PGM sequencing. MiSeq libraries were prepared from 1 ng of PCR product using the Nextera XT library preparation kits, and sequencing was performed using the MiSeq 300 cycle v2 kits as per the manufacturer’s instructions including on-instrument cluster generation. Ion Torrent libraries were prepared using 100 ng of each product in combination with the Ion Xpress Plus Library Kit for AB Library Builder. Library Fragments were clonally amplified onto Ion Sphere particles by emulsion PCR using the Ion One Touch Template Preparation kits, and sequenced on Ion Torrent 318 chips. In both cases libraries were pooled prior to sequencing and results obtained within 24 hours of the initial PCR reactions.

#### Informatics Pipeline

SNP arrays were analysed with Illumina BeadStudio, PLINK and a set of custom scripts. Exome and targeted mitochondrial sequencing reads were aligned to the reference human genome (hg19) using the Burrows-Wheeler alignment (BWA) and down-stream processing of sequence data was done with Picard v1.8, SAMtools v0.1.18 and the Genome Analysis Toolkit (GATK) version 2.2.8. Variants (SNP and Indels) were identified using GATK, following version four of the GATK Best Practice Variant Detection guide. Variants were annotated using Annovar with UCSC Known Genes models, and known polymorphisms were identified using dbSNP135, 1000 genomes and NHLBI exome project with minor allele frequencies recorded from each dataset. Subsequent analysis and identification of candidate variants was performed using an in-house workflow incorporating the annotated variant data and pedigree information.

### Spectrophotometric assays of the respiratory chain enzyme activity

Spectrophotometric determinations of enzyme activity for all respiratory chain enzymes from skeletal muscle and liver biopsies were performed as described previously [10].

### qPCR quantification of mutant load

PCR primers specific to the *MT-ND3* (m.10134C>A) mutation, and to wild-type controls were designed and are available upon request. Quantitative PCR was performed using SYBR green dye and 1 Unit of Immolase DNA Polymerase (Bioline, Alexandria, NSW, Australia), with a final concentration of 1.5 mM MgCl_2_, 500 pM of each primer, 2% DMSO and 1 M betaine. PCR products were generated from 5 ng of genomic DNA isolated (Qiamp DNA Mini kit) from the proband’s blood, fibroblasts, muscle and liver, and from the blood of all other family members. Standard curves were generated from ten-fold dilutions of the mutant and wildtype amplicons inserted into pCR^2.1^TOPO vector (Life Technologies). The cycle conditions used were: 95°C for 12 minutes, (95°C for 10 s, 66°C for 15 s, 72°C for 10 s)×35 cycles.

### Western Blot

Tissue samples were lysed as previously described [11], and whole cell lysates from fibroblasts (15 µg of protein), and from muscle and liver (5 µg of protein), were separated by SDS-PAGE on a 4–12% polyacrylamide gel. A PVDF membrane was probed for 2 h at RT with 1∶1,000 MitoProfile Total OXPHOS Human WB Antibody Cocktail (ab110411, Abcam). Anti-mouse IgG HRP (GE Healthcare) at 1∶2,500 was used as the secondary antibody. Membranes were developed with ECL and exposed on Hyperfilm. Films were scanned on a Microtek ScanMaker 8700 and analysed using ImageQuant (GE Healthcare).

## Results

In this case of a patient with typical LS features we undertook a tiered investigation of increasing resolution that consisted of (i) high-density SNP arrays, (ii) exome sequencing, (iii) targeted ultra-deep mitochondrial sequencing on two different platforms, and (iv) targeted multi-tissue sequencing. Analysis of the SNP array data revealed no potentially pathogenic copy number or genomic rearrangements. Exome sequencing was performed at high depth - a total of 71 gigabases were generated from a single rapid HiSeq 2500 sequencing run, yielding a non-redundant depth of 69x with 96% of targeted bases covered at 18-fold or greater. Both an unbiased genome-wide screen of all nuclear encoded genes and a manual investigation of known nuclear-encoded mitochondrial disease associated genes, however, failed to reveal any putatively disease causing homozygous, compound heterozygous or *de* *novo* mutations.

Taking advantage of the high-depth exome sequencing, we used off-target mitochondrial genome sequencing reads to survey for putative disease-causing variants and identified a low-coverage (6x) m.10134C>A mutation that was unique to the proband. This variant induces a p.Gln26Lys change in *MT-ND3* (NADH-ubiquinone oxidoreductase chain 3), a gene that has previously been associated with LS [12]–[14]. The mutation (NC_012920.1:m.10134C>A; ClinVar SCV000153651) was predicted to be damaging by SIFT with an annotation of “Deleterious” with a score of −3.800, and “Possibly damaging” by PolyPhen2 with a score of 0.611 [15], [16]. The m.10134C>A variant is not recorded in dbSNP, was absent from the mtDB database, which lists mtDNA variants in 2704 sequenced mtDNA genomes [17], and was not detected in any of over 300 control exomes previously sequenced by our group.

We performed targeted ultra-deep mitochondrial investigations of the m.10134C>A mutation on the both the MiSeq (∼20,000-fold) and the Ion Torrent (∼5,000-fold), which revealed that the father and all three unaffected siblings carried only the wild-type allele, and that both the proband and the mother carried the m.10134C>A allele at levels of ∼99.9% and 1% respectively (Table 1). These data strongly indicated that the p.Gln26Lys nonsynonymous *MT-ND3* change was likely to be the cause of LS in this patient.

**Table 1:** Targeted mitochondrial next-generation sequencing.

	MiSeq					Ion Torrent				
	A	C	G	T	Indel	A	C	G	T	Indel
Proband										
- blood	18,093	7	16	11	8	4,373	99	1	0	1638
- fibroblasts	2,168	15	9	11	2	-	-	-	-	-
- liver	2,358	11	11	11	1	-	-	-	-	-
- muscle	2,658	14	17	14	2	-	-	-	-	-
Mother	168	19,842	3	8	0	24	12,217	0	18	262
Father	7	22,999	5	16	0	1	15,010	1	16	248
Sib1	9	20,636	0	18	1	1	4,940	1	15	80
Sib2	13	25,118	3	17	2	1	6,965	0	21	140
Sib3	11	21,979	1	11	4	5	13,813	1	22	277

To assess the functional consequences of the m.10134C>A (p.Gln26Lys) mutation on *MT-ND3*, spectrophotometric enzyme assays of Complex I were performed, which revealed that Complex I was 24%, 17% and 13% of normal relative to protein, citrate synthase and Complex II, respectively, in patient skeletal muscle; and that Complex I was low relative to CS (50%) and Complex II (34%), but normal relative to protein in liver. Additionally, Complex II, citrate synthase and Complex IV activities were elevated in both muscle and liver, and Complex III was elevated in liver (Table 2), suggesting some degree of compensatory mitochondrial proliferation. Analysis by Western blot mirrored the enzyme assay results, showing a reduction in Complex I protein levels in muscle, but normal levels in liver (Figure 1D).

**Table 2:** Muscle and liver respiratory chain enzyme activity.

	Ref Range	Activity (%)	% CS Ratio	% CII ratio
***Muscle***				
Complex I *(nmol/min/mg)*	(19–72)	**10 (24)**	**17**	**13**
Complex II *(nmol/min/mg)*	(26–63)	81 (180)	123	-
Complex III *(/min/mg)*	(12.8–50.9)	40.5 (139)	92	76
Complex IV *(/min/mg)*	(3.3–9.1)	19.8 (300)	209	170
Citrate Synthase *(nmol/min/mg)*	(85–179)	187 (145)	-	-
***Liver***				
Complex I *(nmol/min/mg)*	(8–11)	7 (74)	50	34
Complex II *(nmol/min/mg)*	(54–73)	134 (220)	147	-
Complex III *(/min/mg)*	(5.2–10.3)	23.7 (312)	208	143
Complex IV *(/min/mg)*	(0.5–0.9)	2.44 (344)	233	157
Citrate Synthase *(nmol/min/mg)*	(26–31)	41 (146)	-	-

Note: Bold characters indicate diagnostically abnormal values. Complex I, NADH-coenzyme Q1 oxidoreductase; Complex II, succinate-coenzyme Q1 oxidoreductase; Complex III, decylbenzylquinol-cytochrome c oxidoreductase; Complex IV, cytochrome *c* oxidase. Data are expressed as a ratio relative to citrate synthase (% CS ratio) and Complex II activity (% CII ratio).

To investigate the tissue-specific load of the m.10134C>A mutation in the proband, ultra-deep targeted MiSeq sequencing and qPCR of DNA from muscle, liver and fibroblasts was performed. Sequencing revealed homoplasmy for the mutation in all tissues tested (Table 1), which was confirmed by qPCR. This suggests that the differences in relative Complex I activity in muscle and liver are not the result of developmental mitochondrial mosaicism, and may simply reflect patient- and tissue-specific variability on the impact of the mutation on complex I enzyme activity, as noted for another *MT-ND3* mutation, m.10191T>C [13]. These data also highlight the fact that homoplasmic mitochondrial mutations are not by definition embryonic lethal, and may have variable and tissue-specific consequences which are likely to be tied to background genetics (see below).

## Discussion

To resolve the genetics underpinning LS in this case we employed a tiered investigation using technologies of increasing resolution that resulted in the identification of a novel mutation in *MT-ND3* (m.10134C>A, p.Gln26Lys), which facilitated patient inclusion into the phase 2B clinical trial of EPI-743. The SNP arrays, exome and targeted mitochondrial ultra-deep sequencing were completed in less than three and a half weeks, a timeframe comparable to commercially available single gene diagnostic tests. Other orthogonal approaches may have resolved the case in a similar timeframe (e.g. Sanger sequencing of the mitochondrial genome partnered with targeted nuclear gene investigations), but we note that the approach used here provided both maximum depth and breadth and therefore the highest likelihood of causal mutation detection. Access to genetic material from all members of the immediate family virtually eliminated all false positives. Amongst nuclear encoded genes we found only one putative *de novo* mutation, and no homozygous or compound heterozygous mutations that segregated with the proband, thus reducing the overall period of the investigation.

Three other LS *MT-ND3* mutations have been reported - m.10158T>C, m.10191T>C, m.10197G>A [12]–[14] - and may shed light on some of the results described here. For example, a recent meta-analysis of patients carrying the m.10191T>C mutation demonstrated that there is no clear correlation between mutant load and Complex I activity in muscle and liver [3]. This is consistent with the observed marked decrease in Complex I activity in muscle, but little overall reduction in liver, despite the fact that both tissues are homoplasmic by both qPCR and targeted next-generation sequencing for the m.10134C>A mutation. The biology underpinning such tissue specific variation in enzyme activities and compensatory responses is unresolved, but is likely to be linked to the mutation’s affect on MT-ND3 activity in the context of a specific molecular background. Indeed, prior work investigating m.10191T>C has shown a reduction in Complex I catalytic activity [13], which is consistent with the results presented here that show a reduction in enzyme activity (Table 2) while protein levels are relatively unaffected (Figure 1D). Higher residual activity of Complex I in liver compared with muscle is not unique to *MT-ND3* mutations. We have found similar differences with residual activity of Complex I being 2- to 5-fold higher in liver than muscle for patients with near-homoplasmic *MT-ND6* mutations and in patients with autosomal recessive mutations in the *NDUFS6*, *NDUFS8* and *NDUFV1* Complex I subunit genes (unpublished data). Similarly, there does not appear to be a simple correlation as to why Complex I subunit mutations appear to induce mitochondrial proliferation in some tissues and patients but not others. While it would be interesting to study markers of mitochondrial biogenesis in patient biopsies, this is usually impractical due to the limitation in size of biopsies, at least for paediatric patients.

The clinical utility of an un-constrained high-throughput sequencing approach, particularly in cases with an unresolved diagnosis, is now clearly established. It is also evident, that the approaches used in such cases must be largely free of systematic biases. We note that in our study that the m.10134C>A mutation created a four base adenine homopolymer (Figure 1C) that resulted in a 27% indel call rate in the proband’s Ion Torrent targeted sequencing data, and masked the low-level mutation in the maternal sample (Table 1). This highlights the need for multiple validated and orthogonal sequencing approaches in clinical and diagnostic investigations.

We note that identification of the causal mutation in the proband was successful despite the initial dependence on low-coverage off-target mitochondrial sequencing reads, and potential issues stemming from nuclear copies of mitochondrial DNA (NUMTs), suggesting that next-generation sequencing approaches may be particularly useful in the diagnosis of mitochondrial disorders. This is likely to be particularly true if more targeted, e.g. directed mtDNA sequencing, or broader approaches, e.g. whole genome sequencing (WGS), as opposed to WES, are employed. Indeed, although WGS is currently cost-prohibitive for most research and diagnostic investigations, its use would have dramatically reduced the time required to resolve this case. Whole genome sequencing libraries can be generated in less than 24 hours, and running all six family members would have taken a little more than a week and yielded at least 1,000x coverage of the mitochondrial genome. We anticipate that as sequencing costs continue to fall the utility of WGS for resolution of genetically mediated mitochondrial disease will increase. Consistent with previous reports [18]–[20], in this case we observed ultra-low level maternal heteroplasmy of the m.10134C>A mutation and homoplasmy in the proband, underscoring the importance of using high-throughput strategies for mitochondrial disease gene identification in apparently sporadic cases.

## References

[1] Baertling F, Rodenburg RJ, Schaper J, Smeitink JA, Koopman WJ, et al.. (2013) A guide to diagnosis and treatment of Leigh syndrome. J Neurol Neurosurg Psychiatry: 1–9. 10.1136/jnnp-2012-304426 23772060

[2] Finsterer J (2008) Leigh and Leigh-like syndrome in children and adults. Pediatr Neurol 39: 223–235. 18805359 10.1016/j.pediatrneurol.2008.07.013

[3] Nesbitt V, Morrison PJ, Crushell E, Donnelly DE, Alston CL, et al (2012) The clinical spectrum of the m.10191T>C mutation in complex I-deficient Leigh syndrome. Dev Med Child Neurol 54: 500–506. 22364517 10.1111/j.1469-8749.2012.04224.x

[4] Thorburn DR, Rahman S (2003) Mitochondrial DNA-Associated Leigh Syndrome and NARP. In: Pagon RA, Adam MP, Bird TD, Dolan CR, Fong CT et al.., editors. GeneReviews. Seattle (WA). 1–9.

[5] Goldstein AC, Bhatia P, Vento JM (2013) Mitochondrial disease in childhood: nuclear encoded. Neurotherapeutics 10: 212–226. 23516041 10.1007/s13311-013-0185-6 PMC3625393

[6] Distelmaier F, Koopman WJ, van den Heuvel LP, Rodenburg RJ, Mayatepek E, et al (2009) Mitochondrial complex I deficiency: from organelle dysfunction to clinical disease. Brain 132: 833–842. 19336460 10.1093/brain/awp058

[7] Rahman S, Blok RB, Dahl HH, Danks DM, Kirby DM, et al (1996) Leigh syndrome: clinical features and biochemical and DNA abnormalities. Ann Neurol 39: 343–351. 8602753 10.1002/ana.410390311

[8] Martinelli D, Catteruccia M, Piemonte F, Pastore A, Tozzi G, et al (2012) EPI-743 reverses the progression of the pediatric mitochondrial disease–genetically defined Leigh Syndrome. Mol Genet Metab 107: 383–388. 23010433 10.1016/j.ymgme.2012.09.007

[9] Maitra A, Cohen Y, Gillespie SE, Mambo E, Fukushima N, et al (2004) The Human MitoChip: a high-throughput sequencing microarray for mitochondrial mutation detection. Genome Res 14: 812–819. 15123581 10.1101/gr.2228504 PMC479107

[10] Frazier AE, Thorburn DR (2012) Biochemical analyses of the electron transport chain complexes by spectrophotometry. Methods Mol Biol 837: 49–62. 22215540 10.1007/978-1-61779-504-6_4

[11] Cooper ST, Kizana E, Yates JD, Lo HP, Yang N, et al (2007) Dystrophinopathy carrier determination and detection of protein deficiencies in muscular dystrophy using lentiviral MyoD-forced myogenesis. Neuromuscul Disord 17: 276–284. 17303423 10.1016/j.nmd.2006.12.010

[12] Crimi M, Papadimitriou A, Galbiati S, Palamidou P, Fortunato F, et al (2004) A new mitochondrial DNA mutation in ND3 gene causing severe Leigh syndrome with early lethality. Pediatr Res 55: 842–846. 14764913 10.1203/01.PDR.0000117844.73436.68

[13] McFarland R, Kirby DM, Fowler KJ, Ohtake A, Ryan MT, et al (2004) De novo mutations in the mitochondrial ND3 gene as a cause of infantile mitochondrial encephalopathy and complex I deficiency. Ann Neurol 55: 58–64. 14705112 10.1002/ana.10787

[14] Sarzi E, Brown MD, Lebon S, Chretien D, Munnich A, et al (2007) A novel recurrent mitochondrial DNA mutation in ND3 gene is associated with isolated complex I deficiency causing Leigh syndrome and dystonia. Am J Med Genet A 143: 33–41. 10.1002/ajmg.a.31565 17152068

[15] Choi Y, Sims GE, Murphy S, Miller JR, Chan AP (2012) Predicting the functional effect of amino acid substitutions and indels. PLoS One 7: e46688. 23056405 10.1371/journal.pone.0046688 PMC3466303

[16] Adzhubei IA, Schmidt S, Peshkin L, Ramensky VE, Gerasimova A, et al (2010) A method and server for predicting damaging missense mutations. Nat Methods 7: 248–249. 20354512 10.1038/nmeth0410-248 PMC2855889

[17] Ingman M, Gyllensten U (2006) Human Mitochondrial Genome Database, a resource for population genetics and medical sciences. Nucleic Acids Res 34: D749–D751. 16381973 10.1093/nar/gkj010 PMC1347373

[18] Thorburn DR (2004) Mitochondrial disorders: prevalence, myths and advances. J Inherit Metab Dis 27: 349–362. 15190193 10.1023/B:BOLI.0000031098.41409.55

[19] Swalwell H, Kirby DM, Blakely EL, Mitchell A, Salemi R, et al (2011) Respiratory chain complex I deficiency caused by mitochondrial DNA mutations. Eur J Hum Genet 19: 769–775. 21364701 10.1038/ejhg.2011.18 PMC3137493

[20] Tarnopolsky M, Meaney B, Robinson B, Sheldon K, Boles RG (2013) Severe infantile leigh syndrome associated with a rare mitochondrial ND6 mutation, m.14487T>C. Am J Med Genet A 161A: 2020–2023. 23813926 10.1002/ajmg.a.36000

